# RETRACTION: Assessing the Role of Combination of Stem Cell and Light‐Based Treatments on Skin Wound Repair: A Meta‐Analysis

**DOI:** 10.1111/iwj.70226

**Published:** 2025-02-11

## Abstract

**RETRACTION**: ZhengM. , ZhangH. , WuH. , XieJ. , ChenQ. , JiangY. , and ZhaoD. , “Assessing the Role of Combination of Stem Cell and Light‐Based Treatments on Skin Wound Repair: A Meta‐Analysis,” International Wound Journal 20, no. 10 (2023): 4272–4280, 10.1111/iwj.14329.37525509 PMC10681544

The above article, published online on 31 July 2023, in Wiley Online Library (http://onlinelibrary.wiley.com/), has been retracted by agreement between the journal Editor in Chief, Professor Keith Harding; and John Wiley & Sons Ltd. Following an investigation by the publisher, all parties have concluded that this article was accepted solely on the basis of a compromised peer review process. The editors have therefore decided to retract the article. The authors did not respond to our notice regarding the retraction.

**RETRACTION**: M. Zheng , H. Zhang , H. Wu , J. Xie , Q. Chen , Y. Jiang , and D. Zhao , “Assessing the Role of Combination of Stem Cell and Light‐Based Treatments on Skin Wound Repair: A Meta‐Analysis,” International Wound Journal 20, no. 10 (2023): 4272–4280, 10.1111/iwj.14329.37525509 PMC10681544

The above article, published online on 31 July 2023, in Wiley Online Library (http://onlinelibrary.wiley.com/), has been retracted by agreement between the journal Editor in Chief, Professor Keith Harding; and John Wiley & Sons Ltd. Following an investigation by the publisher, all parties have concluded that this article was accepted solely on the basis of a compromised peer review process. The editors have therefore decided to retract the article. The authors did not respond to our notice regarding the retraction.

